# Understanding the unique mechanism of ferroptosis: a promising therapeutic target

**DOI:** 10.3389/fcell.2023.1329147

**Published:** 2024-03-18

**Authors:** Yuanyuan Kong, Jing Li, Rufeng Lin, Shifeng Lu, Liucheng Rong, Yao Xue, Yongjun Fang

**Affiliations:** ^1^ Department of Hematology and Oncology, Children’s Hospital of Nanjing Medical University, Nanjing, China; ^2^ Department of Clinical Laboratory, Maternal and Child Health Care of Zaozhuang, Zaozhuang, China

**Keywords:** ferroptosis, cell death, iron metabolism, reactive oxygen species, GPX4

## Abstract

Ferroptosis is an iron-dependent form of regulated cell death and is characterized by high concentrations of intracellular lipid peroxide and a redox imbalance in the cells. Ferroptosis shows distinct morphological and biological features compared with other prominent mechanisms of programmed cell death. The distinct characteristics of ferroptosis include the dysfunction of the lipid peroxide repair enzyme glutathione peroxidase 4, the presence of ferrous iron overload, and the lipid peroxidation of polyunsaturated fatty acids. Several other metabolic pathways (including iron, lipid, and amino acid metabolism) and ferritinophagy, as well as transcription factors, can modulate ferroptosis. However, to date, the molecular mechanism of ferroptosis has not been elucidated. This review outlines the discovery, characterization, regulatory mechanisms, and crosstalk of ferroptosis. Further, we have noted the controversial elements in the ferroptosis-related mechanisms. Our inferences may provide a partial reference for developing strategies to regulate ferroptosis.

What is already known on this topic—Ferroptosis include the dysfunction of the lipid peroxide repair enzyme glutathione peroxidase 4, the presence of ferrous iron overload, and the lipid peroxidation of polyunsaturated fatty acids.
What this study adds—Outlines the discovery, characterization, regulatory mechanisms, and crosstalk of ferroptosis.
How this study might affect research, practice or policy –May provide a partial reference for developing strategies to regulate ferroptosis.

## Introduction

Cell death is an integral cellular process that occurs in living cells (Doll and Conrad, 2017). The process maintains homeostasis and prevents certain hyperproliferative diseases such as cancer (Fuchs and Steller, 2015). Under certain conditions, dead cells are continuously replaced by new ones causing aberrant cell growth in the diseased condition. Apart from accidental cell death that occurs suddenly after extremely harsh physicochemical or mechanical insults, most cell death types can be regulated by genetic or pharmacologic interferences (regulated cell death) (Galluzzi et al., 2015; Doll and Conrad, 2017). In the early 1970s, the three canonical types of cell death were defined by the structure and morphology of the dying cell; they include apoptosis (type I; physiological cell death), autophagy (type II), and necrosis (type III; pathological cell death). In addition, new non-canonical cell death modalities have been proposed, including paraptosis, pyroptosis, mitotic catastrophe, autophagy-dependent cell death, lysosome-dependent cell death, immunogenic cell death, and ferroptosis (Gilloteaux et al., 2010). Ferroptosis is a non-apoptotic mode of regulatory necrosis with distinct morphologic and biochemical features. The process is distinguished from other programmed and non-programmed cell death types by its iron dependency and intracellular lipid peroxide accumulation (Dixon et al., 2012a).

While ferroptosis was originally discovered in the context of screening for small molecules to selectively kill RAS-mutated tumor cells, its relevance has expanded to other pathologic settings as well. Ferroptosis plays a critical regulatory role in various human diseases, such as tumorigenesis, neurodegenerative diseases, ischemia-reperfusion injury, renal failure, organ fibrosis, cardiovascular diseases, and hematological diseases (Li J. et al., 2020). The initiation of ferroptosis is a desirable outcome in several carcinomas. The efficacy of chemotherapy improved in certain tumor types that were susceptible to modulations in this death pathway (Stockwell et al., 2017). However, ferroptosis is a negative outcome in several other diseases, such as neurodegeneration, type II diabetes, periventricular leukomalacia, and renal dysfunction; it contributes to enhancing injury or disease progression. Therefore, the pathophysiologic and physiologic relevance of ferroptosis in different disease contexts has created several avenues for therapeutic discovery. Herein, we reviewed the different processes involved in the regulation of ferroptosis. This information is crucial in developing novel therapeutic strategies that target ferroptosis for several human diseases, including cancer.

## Overview of ferroptosis

### Discovery

In 1955, human uterine carcinoma HeLa cells that were cultured without cystine revealed a unique microscopic morphology that could be restored by the addition of glutathione (GSH) (EAGLE, 1955). Later, researchers revealed that cystine could maintain the GSH levels and that there might be an accumulation of intracellular reactive oxygen species (ROS) (Bannai et al., 1977). In 1965, lipid peroxidation was considered to be the primary reason for cellular damage in the rat liver. In the 1980s, screening of the unsaturated lipid components of damaged cell membranes identified lipid peroxidation as one of the main forms of oxidative damage (Dolma et al., 2003). As such, these early studies related to the discovery of a novel mechanism of cellular damage.

### Conceptualization

**FIGURE 1:**
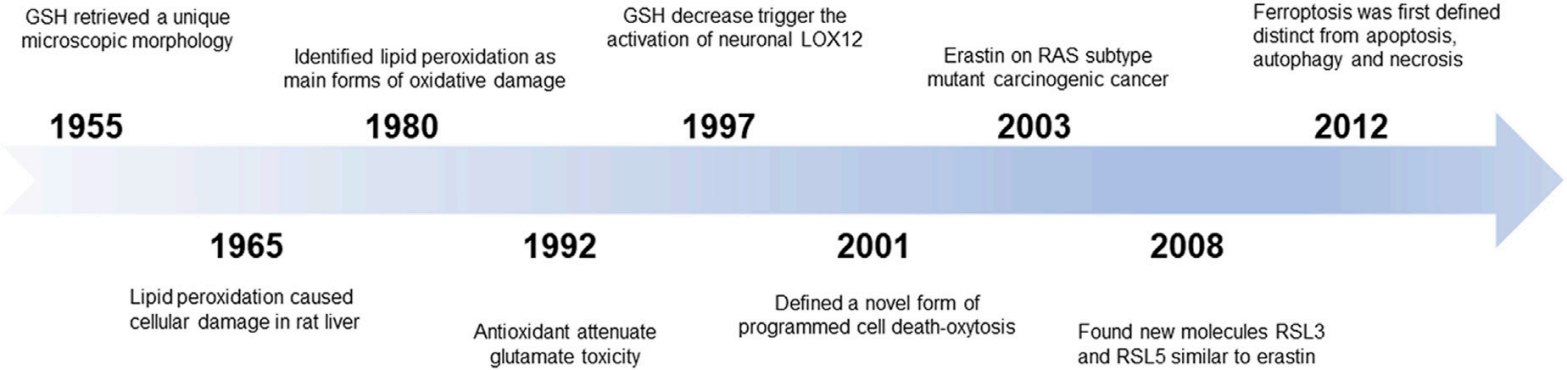
The discovery of ferroptosis.

In 2003, Dolma et al. screened small chemical compounds to test their efficacy to kill cells overexpressing the oncogenic RAS protein (Dolma et al., 2003). This study identified a novel compound named “erastin” that selectively killed RAS-expressing tumorigenic cells in a non-apoptotic manner. In 2007, the authors further confirmed that lipophilic antioxidants (e.g., α-tocopherol) prevented erastin-induced oxidative non-apoptotic death in the HRAS- and KRAS-expressing cells (Dolma et al., 2003). In 2008, two small molecules, RAS-selective lethal molecule 3 and 5 (RSL3 and RSL5), were identified, which selectively killed human foreskin fibroblasts in a similar iron-dependent non-apoptotic manner, and this form of cell death could be inhibited by both desferrioxamine mesylate and α-tocopherol (Yang and Stockwell, 2008). Subsequent studies using other cell death inducers elaborated on the features of this mode of cell death. In 2012, Dixon et al. coined the term “ferroptosis” for this phenomenon of erastin-induced iron-dependent cell death characterized by lipid peroxidation (Dixon et al., 2012a) (Figure 1).

### Characterization

The ferroptotic cells have distinct morphological and biochemical features and phenotypes that distinguish them from cells undergoing other forms of cell death (Table 1). Treatment with a ferroptosis inducer (e.g., erastin) results in small-sized mitochondria with reduced or vanished cristae, condensed mitochondrial densities, and ruptured outer mitochondrial membrane (Dixon et al., 2012a; Xie et al., 2016; Li J. et al., 2020). The biochemical features of ferroptotic cells include accumulation of iron, lipid peroxidation, inhibition of cystine/glutamate antiporter system (system X_c_^−^) and glutathione peroxidase 4 (GPX4), and reduction in cystine uptake and GSH (Dixon et al., 2012a; Xie et al., 2016; Li J. et al., 2020). Six mitochondrial regulator genes specific for ferroptosis, namely, ATP5G3, CS, RPL8, IREB2, TTC35, and ACSF2, were detected using shRNA in HRAS-mutant Calu-1 cells and NRAS-mutant HT-1080 cells (Dixon et al., 2012a). Moreover, the specific ferroptosis inhibitors (such as ferrostatin-1 and deferoxamine) do not affect other types of programmed cell death. However, it is controversial whether the other cell death inhibitors can regulate ferroptosis. Therefore, further studies are required to explore the mechanism of ferroptosis.

**TABLE 1:** Characterization of regulated cell deaths.

Cell death	Morphological features	Biochemical features
Ferroptosis	No rupture of the plasma membrane	Ferrous irons and ROS accumulation
	Rounding up of the cell	Inhibition of system Xc^−^Reduction of cystine and GSH
	Shrunken mitochondria, exterior mitochondrial rupture reduction or absence of the cristate	Activation of MAPKs
	Normal nuclear size and no chromatin condensation	Release of arachidonic acid mediators
Apoptosis	Pyknosis and plasma membrane	Activation of caspases
	Cell shrinkage	PS exposure
	Pseudopod retraction and reduction of cellular and nuclear volume	Oligonucleosomal DNA fragmentation
	Nuclear fragmentation, chromatin condensation	
	Formation of apoptotic bodies	
	No modification of mitochondrial structure	
Necroptosis	Rupture of the plasma membrane	Decrease in ATP wastage
	Swelling of cytoplasm and organelles	Release DAMPs
	Moderate chromatin condensation	PARP1 hyperactivation
	Loss of intracellular organelle	
Autophagy	Lack of change in the plasma membrane	Transformation of MAP1LC3 from LC3Ⅰ to LC3Ⅱ conversion
	Large scale of autophagic vacuoles	Substrate degradation
	Absence of chromatin condensation	
	Formation of double-intracellular vesicles, including macroautophagy, microautophagy and chaperone-mediated autophagy	

*MAPKs*, mitogen-activated protein kinases; *GSH*, glutathione; *DAMPs*, damage-associated molecular patterns; *PS*, phosphatidylserine; *PARP1*, Poly [(ADP-ribose)] polymerase 1; *MAP1LC3*, microtubule-associated protein 1 light chain 3; *LC3I/II*, Microtubule-associated protein light chain 3.

## Regulation mechanisms of ferroptosis

### Iron metabolism

Ferroptosis is an iron-dependent form of regulated cell death. Iron is an important component in several intracellular processes, including DNA synthesis, mitochondrial respiration, cellular respiration, and cell signaling. Iron exists in two forms in the human body, ferrous (Fe^2+^) and ferric (Fe^3+^) ions; the majority of iron is used for metalloprotein synthesis or stored by ferritin to form inorganic (e.g., Fe/S cluster) or organic cofactors (e.g., heme) (Daher et al., 2017). The uptake, transport, storage, and metabolism of iron are precisely regulated because excessive cellular iron [redox-active iron (Fe^2+^)] can increase the production of ROS through the Fenton reaction, thereby promoting lipid peroxidation. Extracellular Fe^3+^ is absorbed from the diet by duodenal villous epithelial cells, binds to transferrin, and is transported by combining with the transferrin receptor (TFR) (Li et al., 2020b). Once internalized, the complex dissolves in the late endosome/lysosome owing to the acidic environment. Free Fe^3+^ is reduced to Fe^2+^ by a six-transmembrane epithelial antigen of prostate 3 metalloreductase (STEAP3) and transferred out of the endosome/lysosome into the cytosol by the apical divalent metal transporter 1 (DMT1) (Li J. et al., 2020). In the cytosol, the acquired iron can be transported to the site of its use. It may also be stored by ferritin as the Fe^3+^ irons or in the transient labile iron pool (LIP) to avoid cell toxicity. The surplus intracellular iron is exported out of the cell by ferroportin (FPN1/SLC40A1) (Kakhlon and Cabantchik, 2002).

High levels of free intracellular iron in the cells are a prerequisite for triggering ferroptosis. Excess cellular iron increases the levels of the Fe^2+^ irons; the resulting increase in the LIP subsequently promotes lipid peroxidation via the Fenton reaction and activity of ROS-generating enzymes which triggers ferroptosis (Hassannia et al., 2019). The iron metabolism genes probably regulate the susceptibility to ferroptosis and its occurrence and development. The most crucial route for cells to import iron is mediated by transferrin and its carrier protein TFR1, which is encoded by the TFR1 gene. Notably, silencing the TFR1 gene inhibited erastin-induced ferroptosis (Kuang et al., 2020). Furthermore, ferroptosis induction or iron-responsive element-binding protein 2 (IREB2) silencing modulates iron content, which is mediated by several genes such as the TFR, ferritin heavy chain, and ferritin light chain genes, rendering the cell more sensitive to ferroptosis (Dixon et al., 2012a). Artemisinin derivatives, which rely on free Fe^2+^ iron to mediate their activity, regulate the expression of iron metabolism genes such as TFR, IREB2, and other specific ferroptosis-related genes. In Hela cells, heat shock factor-binding protein 1 (HSPB1) protects the actin cytoskeleton to obstruct iron uptake by inhibiting the TRF1 gene expression (Sun et al., 2015). In a recent study, excessive expression of iron-sulfur cluster proteins, such as CDGSH iron sulfur domain 1 (CISD1), an iron-containing export mitochondrial membrane protein desensitized tumor cells to ferroptosis (Alvarez et al., 2017). However, the role of mitochondria in ferroptosis remains controversial and further research is needed to explore this phenomenon. Overall, alterations in the expression of iron metabolism genes or inhibition of HSPB1 and CISD1 can significantly lower the free intracellular iron content and trigger iron overload-induced ferroptosis.

**FIGURE 2:**
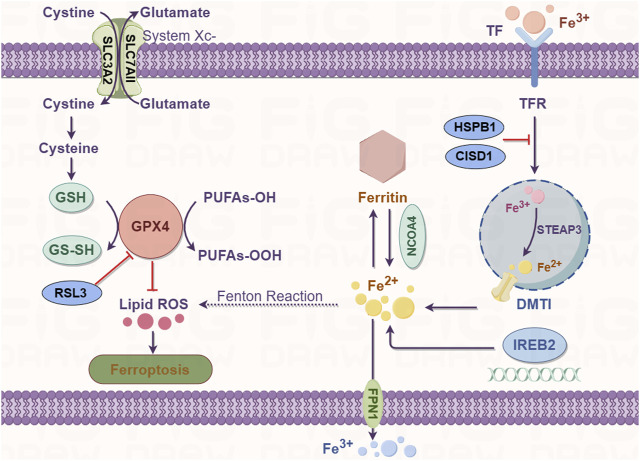
Iron metabolism (right) and GSH/GPX4 (left) pathway diagram.

Ferritinophagy is the autophagic depletion of ferritin by nuclear receptor coactivator 4 (NCOA4). It is a recently identified process that contributes to iron accumulation and free radical damage (Mancias et al., 2014). Ferritin degradation results in the release of iron, thereby increasing LIP and subsequent cellular toxicity. Ferritinophagy plays a critical role in maintaining iron homeostasis. The autophagic degradation of ferritin via NCOA4-mediated ferritinophagy was found to initiate ferroptosis by increasing the levels of free iron, promoting ROS accumulation, and driving the ferroptotic process in the cytoplasm (Lei et al., 2019). The genetic ablation of NCOA4 inhibits erastin-induced ferroptosis, whereas its overexpression significantly increases sensitivity to ferroptosis in HT-1080 fibrosarcoma and PANC1 pancreatic adenocarcinoma cell lines (Hou et al., 2016). Therefore, NCOA4-mediated ferritin degradation is closely related to ferroptosis. Overall, these studies suggest that free intracellular iron levels are tightly regulated and dysregulation of iron metabolism is a key trigger of ferroptosis (Figure 2).

### Depletion of glutathione and ferroptosis

Ferroptosis is an iron-dependent form of cell death; it occurs through the accumulation of ROS when GSH-dependent lipid peroxide repair systems are compromised. System X_c_^−^ is a disulfide-linked heterodimer comprising SLC3A2 and SLC7A11; it imports one molecule of cystine (the oxidized dimer form of cysteine) in exchange for the equivalent molecule of intracellular glutamate antiporter (Tsurusaki et al., 2019). GSH is an antioxidant comprising glutamate, cysteine, and glycine. GPX4 utilizes GSH as a cofactor and is the only selenoenzyme that catabolizes the reduction of lipid hydroperoxide to alcohol (Lei et al., 2019). Consequently, the inhibition of system X_c_^−^ activity by erastin causes the intracellular depletion and inhibition of GSH levels by decreasing the influx of cysteine. This depletes GPX activity and results in the reduction in cell antioxidant capacity, lethal accumulation of ROS, and initiation of ferroptosis by erastin-induced oxidative stress (Yang et al., 2014).

GPX4 is a monomeric glutathione peroxidase responsible for peroxide detoxification in complex lipids and is the main protector of cells from ferroptotic cell death (Yang and Stockwell, 2008). Deficiency and inhibition of GPX4 induce cell death in a ferroptotic manner (Kim et al., 2020). A decrease in GPX4 activity is mediated by direct or indirect mechanisms (such as GSH depletion). In the modulatory profiling strategy, erastin and RSL3 (the first identified ferroptotic compounds) are xCT antiporter (consisting of two subunits SLC3A2 and SLC7A11) and GPX4 inhibitors, respectively. A mechanism underlying erastin-mediated inhibition of the system X_c_^−^ transporter has been shown to initiate ferroptosis (Dixon et al., 2012a) (Figure 2). Other than diminishing the abundance of GPX4 through GSH depletion, the direct inactivation or depletion of GPX4 is mediated by RSL3 and wathaferin A (Hassannia et al., 2018). The ferroptosis-inducing agent 56 (FIN56) directly inhibits GPX4 activity at the posttranslational level without GSH depletion (Shimada et al., 2016a). Moreover, GPX4 inactivation can be induced by several compounds, such as sorafenib, sulfasalazine, L-buthionine sulfoximine, glutamate, and artesunate (Li et al., 2020c). These compounds are involved in GSH depletion and ROS accumulation in the form of lipid hydroperoxides, ultimately due to oxidative imbalance and ferroptosis.

Recently, several studies on ferroptosis have suggested the mutational or chemical inhibition of the cystine-glutamate antiporter in diverse cancers. Glutamate, by preventing cystine import, could initiate ferroptosis at high extracellular concentrations (Dixon et al., 2012a). Cysteine, imported into cells through the SLC7A10 transporter, is also essential for ferroptotic cell death (Felley-Bosco and Gray, 2019). Some cells synthesize cysteine from methionine through methionine-R-sulfide reductase B2 by trans-sulfuration pathway. This process bypasses the requirement for cystine import via system X_c_^−^ (Stockwell et al., 2017; Felley-Bosco and Gray, 2019). Moreover, the conditional ablation of SLC7A11 initiates tumor-selective ferroptosis, which inhibited pancreatic ductal adenocarcinoma growth (Badgley et al., 2020). Consistent with the above findings, GPX4 inactivation using ferroptosis inhibitors such as ferrostatin-1 and liproxstatin-1 was lethal in xenograft models in many human diseases including acute renal failure, ischemia/reperfusion injury, neurodegenerative diseases, and cancers (Shi et al., 2021). Nicotinamide adenine dinucleotide phosphate (NADPH), which is involved in lipid peroxidation and synthesis of GSH, can promote sensitivity to ferroptosis-inducing agents and is essential for the malfunctioning of GSH metabolism (Shimada et al., 2016b). The nucleophile catalytic moiety of the selenocysteine on the active site of GPX4 plays a pivotal role in these pathologies and the modulation of ferroptosis. GSH is involved in the critical role of maintaining membrane integrity favored by inhibition and impairment of cysteine oxidation and the elimination of electrophiles involved in lipid peroxidation.

### Lipid peroxidation

**FIGURE 3:**
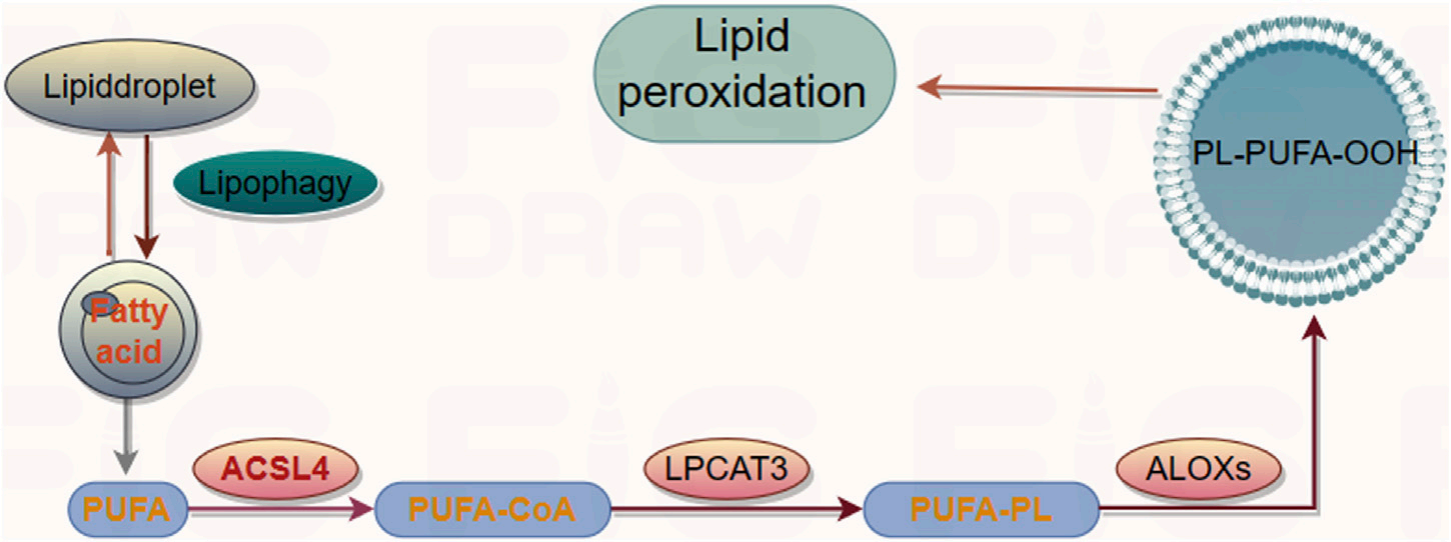
The signal pathway of lipid peroxidation.

Lipid peroxidation is a free radical or non-radical-driven chain reaction that is initiated by the interaction of activated ROS with polyunsaturated fatty acids (PUFAs), which are susceptible to oxygenation to generate lipid peroxyl radicals and hydroperoxides (Friedmann et al., 2014). It occurs in three phases: initiation, propagation, and termination (reviewed in detail in (Yin et al., 2011)). PUFAs, the substrates for iron-catalyzed lipid peroxidation are present in phospholipids. The peroxidation is ultimately catalyzed by lysophosphatidylcholine acyltransferase 3 (LPCAT3) after the acetylation of acyl-CoA synthetase long-chain member 4 (ACSL4) (Dixon et al., 2015). LPCAT3 and ACSL4 are the biomarkers that correlate with the biosynthesis and remodeling of lipid peroxides while executing ferroptosis (Doll et al., 2017) (Figure 3). Lipoxygenases (LOXs) are the other key drivers of lipid peroxidation. LOXs induce the loss of membrane stability and integrity, catalyze the oxidation of PUFAs, and release noxious aldehydes (Hassannia et al., 2019), thereby modulating ferroptosis. The knockdown or inhibition of LOX activity inhibited the occurrence of ferroptosis in some contexts. ROS accumulation is caused by either an increase in their generation or a decrease in their elimination. However, the mechanism by which the ferroptotic death cascade occurs remains unclear. Recent studies on the transcriptional regulation underlying ferroptotic cell death have revealed that several transcription factors (including TP53, NFE2L2/NRF2, YAP1, TAZ, ATF3, ATF4, and HNF4A) can recognize specific DNA sequences to mediate the expression of multiple genes and play multiple roles in determining ferroptosis sensitivity (Dai et al., 2020). These transcription factors may exert their effects through either transcription-dependent or -independent mechanisms (Kang et al., 2019). The function of transcription factors generally depends on the targeted genes, and the same regulators sometimes may regulate both ferroptosis and other cell death forms (Tang et al., 2019). Overall, targeting the ferroptotic network appears to be a new therapeutic strategy for several diseases or pathological conditions.

## Diseases associated with ferroptosis

### Ferroptosis and cancer

**FIGURE 4:**
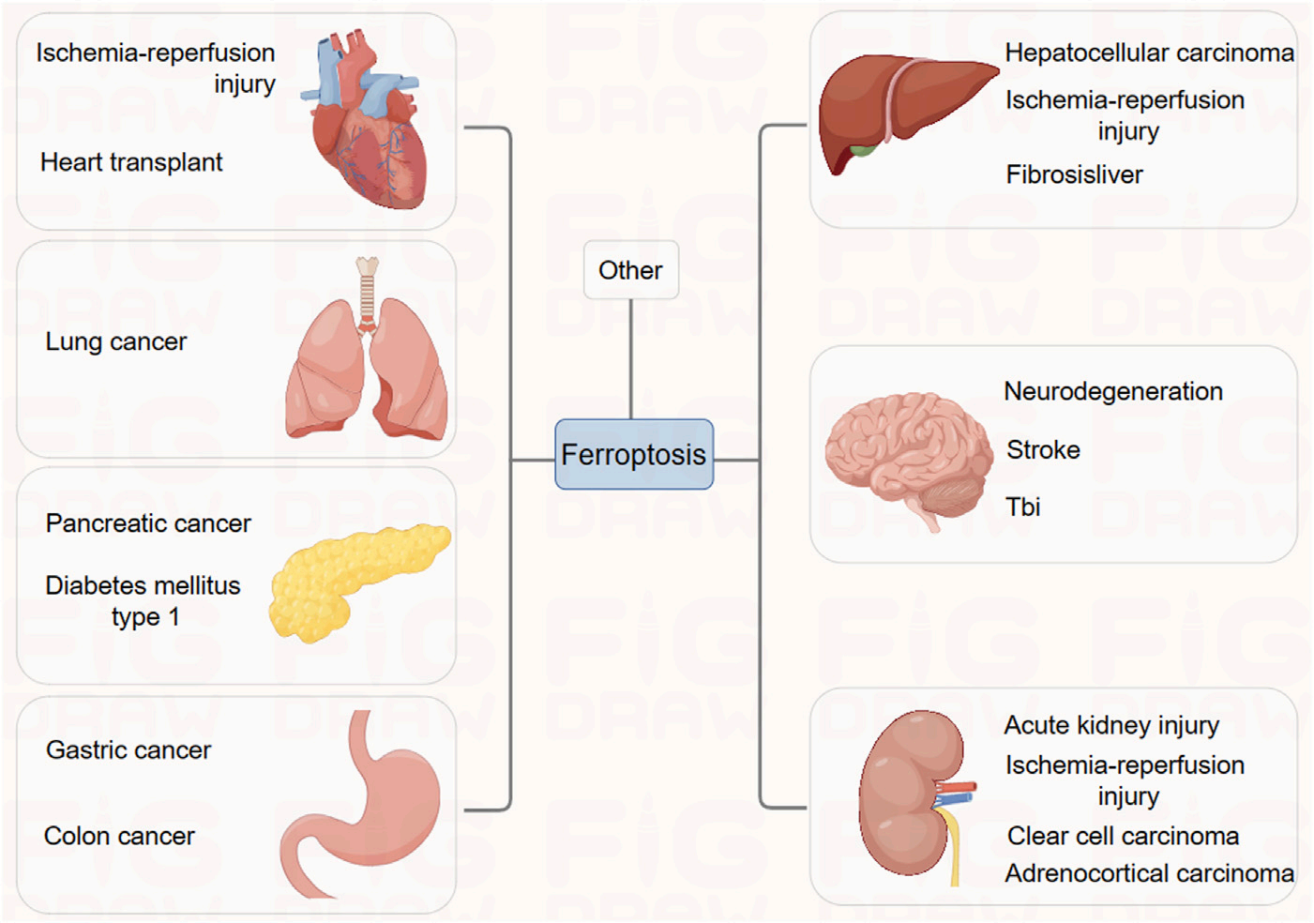
The association between ferroptosis and clinical diseases.

Research suggests that the accumulation of iron ions within tumor cells is closely associated with tumor growth and metastasis in cancer (Figure 4). Excessive accumulation of iron ions may induce oxidative stress in certain types of tumors, leading to apoptosis or necrosis of cancer cells (Chen et al., 2015). Additionally, various tumor suppressor factors have been found to enhance cell sensitivity to ferroptosis, implying that ferroptosis might contribute to the anti-tumor activity of these factors. For instance, studies indicate that p53 promotes ferroptosis by inhibiting the transcription of SLC7A11 (Wang et al., 2016). Furthermore, a particular single nucleotide polymorphism P47S of p53, which is more prevalent in certain African populations, has been associated with increased susceptibility to cancer and resistance of cancer cells to p53 (Jennis et al., 2016). However, further research is needed to explore other functions of these specific mutations in addition to impairing the pro-ferroptotic activity of p53. Moreover, tumor suppressor factor BAP1, an epigenetic regulator, has also been shown to downregulate SLC7A11 expression to promote ferroptosis, but the extent of BAP1’s pro-ferroptotic activity in tumor suppression remains unclear (Zhang et al., 2018).

### Ischemia-reperfusion injury and iron death

Ischemia-reperfusion injury (IRI) results in widespread cell death and inflammatory responses in affected organs, leading to severe conditions such as stroke, ischemic heart disease, liver, and kidney damage. Ischemic heart disease remains one of the deadliest diseases globally.

In studies related to the brain and heart, ferroptosis plays a crucial role in neurotoxicity and brain injury, suggesting that inhibiting ferroptosis might have therapeutic potential. Experiments *in vitro* have demonstrated that ferroptosis inhibitor, ferrostatin-1, can block glutamate-induced neurotoxic cell death (Lau and Tymianski, 2010). As glutamate-induced neurotoxicity is associated with conditions like stroke and various neurodegenerative diseases, and high extracellular glutamate concentrations induce ferroptosis by inhibiting the Xc-system, it suggests that ferroptosis might be one of the pathological mechanisms in these brain-related diseases (Dixon et al., 2012b). Genetic studies have shown that conditional deletion of GPX4 in mice leads to symptoms resembling neurodegenerative diseases. Iron chelators and lipophilic radical-trapping antioxidants (RTAs) have been used in various experimental systems to test their effectiveness in improving stroke and neurodegeneration (Wirth et al., 2010; Chen et al., 2015; Hambright et al., 2017). While systemic use of iron chelators poses serious side effects, these studies confirm the importance of ferroptosis in these diseases.

Regarding ischemic heart disease, extensive research has been conducted on the role of ferroptosis. Studies indicate that in an *ex vivo* system simulating ischemia-reperfusion injury in mouse hearts, iron ion chelators and glutamine breakdown inhibitors significantly reduce myocardial cell death, alleviate cardiac tissue damage, and improve its function (Gao et al., 2015). This suggests the potential value of targeting ferroptosis for treating ischemic heart disease, a view supported by recent *in vivo* studies in mouse models. Designing effective ferroptosis-targeted therapies for acute diseases like stroke and ischemic heart disease requires prompt application, rapid efficacy, and the development of specific inhibitors to minimize side effects on other organs.

Furthermore, in ischemia-reperfusion injury associated with organ transplantation, ferroptosis appears to particularly affect the kidneys (Gao et al., 2015). Survival of proximal renal tubular cells depends on functional GPX4, making these cells highly susceptible during kidney transplantation-associated IRI. Studies have demonstrated that ferroptosis inhibitors alleviate renal tubular cell death and acute kidney failure in models of renal tubular cell IRI in mice, GPX4 knockout mice, and folate-induced acute kidney injury models (Friedmann Angeli et al., 2014; Linkermann et al., 2014). The liver and heart are also associated with organ transplantation (Gao et al., 2015). Overall, ferroptosis inhibitors may contribute to effective treatments for successful organ transplantation across various organs. These findings suggest that inhibiting ferroptosis could be a potential therapeutic method for treating IRI-related diseases.

## Significance and outlook: regulating ferroptosis in tumor immunotherapy

Given the complexity of ferroptosis and its direct impact on various metabolic pathways, it is highly probable that the immune system targets certain crucial steps within the ferroptotic process. In the realm of combining immunotherapy with cysteine deprivation, research suggests that CD8^+^ T cells activated by anti-PD-L1 immunotherapy secrete IFN-γ, promoting ferroptosis in tumor cells post PD-L1 blockade (Wang et al., 2019). IFN-γ secretion notably downregulates the expression of SLC3A2 and SLC7A11 genes in tumor cells, leading to reduced cysteine intake, enhanced lipid peroxidation, and subsequent ferroptosis. Therefore, the combined application of cysteine/cystine dioxygenase with anti-PD-L1 immunotherapy might elicit effective anti-tumor immune responses by inducing ferroptosis.

Regarding the combination of immunotherapy with targeted treatments, recent studies highlight overcoming the resistance to anti-PD-L1 therapy through a combined approach involving TYR03 receptor tyrosine kinase (RTK) inhibitors. TYR03 inhibitors facilitate ferroptosis by modulating the expression of ferroptosis-related genes such as SLC3A2, particularly noted in PD-1-resistant tumors (Xu et al., 2021a). In mouse models of breast cancer, inhibiting TYR03 promotes ferroptosis and heightens the sensitivity of tumors to PD-1 therapy. This research indicates that disrupting ferroptosis via TYR03 inhibitors might be an effective strategy to overcome immunotherapy resistance.

Additionally, evidence suggests a synergistic effect between immunotherapy and radiotherapy (Sun et al., 2018), where radiotherapy enhances sensitivity to ferroptosis. Radiation has been shown to induce ferroptosis by inducing ROS production and upregulating ACSL4, enhancing lipid synthesis, lipid peroxidation, and subsequent membrane damage (Lei et al., 2020; Ye et al., 2020; Lei et al., 2021). The combination of radiotherapy and immunotherapy downregulates SLC7A11 gene expression, mediated by ATM and IFN-γ induced by DNA damage, resulting in reduced cysteine intake, increased ferroptosis, and enhanced tumor control (Jiang et al., 2021). These studies reveal the significance of ferroptosis in the synergistic effects of immunotherapy and radiotherapy.

Finally, immunotherapy might also combine with inhibitors of T-cell ferroptosis, as the ferroptosis of T cells themselves might weaken their immune responses (Xu et al., 2021a; Xu H. et al., 2021). T cells lacking GPX4 accumulate membrane lipid peroxides, leading to ferroptosis. Increased expression of CD36 in tumor-infiltrating CD8^+^ T cells might induce lipid peroxidation, causing functional impairments in these T cells (Xu et al., 2021c; Ma et al., 2021). These findings underscore CD8^+^ T cell ferroptosis as a new mode of tumor immune suppression, highlighting the potential therapeutic benefit of blocking CD36 to enhance anti-tumor immunity. However, GPX4 inhibitors might adversely affect T cells in inducing ferroptosis, leading to unnecessary targeted effects and toxicities.

These latest findings underscore the importance of ferroptosis in immunotherapy and how modulating ferroptosis can enhance or diminish the effectiveness of immunotherapy. Considering the role of ferroptosis in immunotherapy holds significant clinical implications in devising strategies for tumor treatment.

## Conclusion and perspectives

Since its discovery in 2012, ferroptosis has become a promising therapeutic target in cancer research. Several researchers are exploring its clinical roles and pathogenesis, and new ferroptosis-inducing agents are being used for therapy. Ferroptosis is a novel mode of iron-dependent programmed cell death, which is induced by small molecules such as erastin and RSL3 (Table 2). The process is regulated by multiple metabolic pathways such as iron metabolism, GSH metabolism and lipid metabolism, and is accompanied by lethal ROS accumulation, which leads to lipid peroxidation of the cell membrane. However, specific molecular markers for ferroptosis are not available, and the relevant mechanisms need to be further studied. This unique cell death pattern has generated numerous chemotherapeutic possibilities. However, the molecular mechanism of ferroptosis in cancer and its association with other diseases remains to be clarified. Several questions are unanswered in this context. For example, are there other pathways of ferroptosis regulation in addition to the classical pathway? Which is the main pathway that catalyzes the generation of lipid peroxides, the nonenzymatic or enzymatic pathway? Is there any mechanism of ferroptosis resistance? What are the molecular targets of ferroptosis-inducing therapy? How can ferroptosis-inducing antitumor drugs be used in clinical practice? Therefore, further research is required to comprehensively explore the mechanism of ferroptosis for eliminating aggressive tumors.

**TABLE 2:** Based on different mechanisms, currently known iron-induced cell death inducers can be broadly categorized into four types: Class I: Inhibitors of Xc-system; Class II: Inhibitors or degraders of GPX4; Class III: Depleters of coenzyme Q10; Class IV: Inducers of lipid peroxidation via iron or polyunsaturated fatty acid (PUFA) overload. These four types exhibit high specificity in inducing iron-dependent cell death, meaning that during the induction of iron-induced cell death, there is minimal activation of other types of cell death markers.

Type	Ferroptosis inducers	Mechanism of action
I	Erastin and derivative	Inhibition of cystine uptake
	Kinnofen	Inhibition of thioredoxin reductase activity
	Azosulfamide pyridine	Inhibition of cysteine uptake
	Acetaminophen	Depletes intracellular glutathione
	L-buthionine-(S,R)-sulfoximine	Inhibits intracellular glutathione biosynthesis
	Glutamate salt	Reduces Xc- activity by inhibiting glutamate efflux
II	RSL3	Binding to the selenocysteine active site of GPX4
	FIN56	Induce degradation of GPX4
	ML162	Binding of GPX4 to the selenocysteine active site
	ML210	Binding of GPX4 to the selenocysteine active site
	DPI10	Inhibition of GPX4 activity
	DPI13	Binding to GPX4 at other sites
	Altertamine	Inhibition of GPX4 activity
	Cisplatin	Binding to GSH, causing GPX4 inactivation
III	iFSP1	Inhibiting FSP1 activity, reducing coenzyme Q10 generation
	Statin drugs	Inhibit the mevalonate pathway
IV	Heme	Augment the labile iron pool within cells
	Artemisinin	Inducing ferritinophagy to release labile iron pool
	Artesunate	Inducing ferritinophagy to release labile iron pool
	Paratinib	Increase ferritin expression
	FINO2	Oxidize ferrous ions, promote ROS accumulation, oxidize PUFA
	BAY87-2243	Inhibit mitochondrial respiratory chain complex I, increase ROS accumulation
	tBOOH	Oxidation of PUFA

Taken together, multi-directional insights into the underlying regulatory mechanism of ferroptosis have great significance in understanding the pathogenesis, preventing the diseases, and designing therapeutic interventions. Therefore, new studies dedicated to the fundamental research of ferroptosis should be encouraged. The crosstalk between ferroptosis and other forms of cell deaths is another potential research area to determine their cooperation and modulation in cells and tissues. These studies will provide clues for the regulatory mechanism of ferroptosis, and ferroptosis will be a promising therapeutic target in translational medicine.

Five references that we consider to be key references:

1. Dixon SJ, Lemberg KM, Lamprecht MR, Skouta R, Zaitsev EM, Gleason CE, Patel DN, et al. Ferroptosis: an iron-dependent form of nonapoptotic cell death. CELL 2012, 149: 1060-1072
2. Bannai S, Tsukeda H, Okumura H. Effect of antioxidants on cultured human diploid fibroblasts exposed to cystine-free medium. Biochem Biophys Res Commun 1977, 74: 1582-1588
3. Alvarez SW, Sviderskiy VO, Terzi EM, Papagiannakopoulos T, Moreira AL, Adams S, Sabatini DM, et al. NFS1 undergoes positive selection in lung tumours and protects cells from ferroptosis. NATURE 2017, 551: 639-643
4. Yang WS, SriRamaratnam R, Welsch ME, Shimada K, Skouta R, Viswanathan VS, Cheah JH, et al. Regulation of ferroptotic cancer cell death by GPX4. CELL 2014, 156: 317-331
5. Badgley MA, Kremer DM, Maurer HC, DelGiorno KE, Lee HJ, Purohit V, Sagalovskiy IR, et al. Cysteine depletion induces pancreatic tumor ferroptosis in mice. SCIENCE 2020, 368: 85-89
